# Design and Preparation of Molecularly Imprinted Membranes for Selective Separation of Acteoside

**DOI:** 10.3389/fchem.2020.00775

**Published:** 2020-09-29

**Authors:** Xiaobin Zhao, Yun Cheng, Helin Xu, Yanyan Hao, Yin Lv, Xueqin Li

**Affiliations:** Key Laboratory for Green Processing of Chemical Engineering of Xinjiang Bingtuan, School of Chemistry and Chemical Engineering, Shihezi University, Shihezi, China

**Keywords:** molecularly imprinted membrane, acteoside, permeation, enrichment, separation

## Abstract

Acteoside (ACT) belongs to a type of phenylethanoid glycosides (PhGs), and it is worthy of obtaining high-purity due to its significant medicinal functions. In this study, a novel class of MIMs was designed and synthesized with PVDF membranes as the base membrane for high selective separation and enrichment of ACT. The effects of the different functional monomers, the amounts of functional monomers, crosslinking agents, and initiators on the separation properties of MIMs were investigated. Furthermore, adsorption ability, permeation capacity, and reusability of MIMs were discussed for ACT. It indicated that MIM7 was the optimal performance of MIMs. The adsorption ability of MIM7 for ACT was 62.83 mg/g, and the selectivity factor (α) of MIM7 was up to 2.74 and its permeability factor (β) was greater than 2.66. Moreover, the adsorption amount of MIM7 was still more than 88.57% of the initial value after five cycles. As an ACT imprinted layer of MIMs only had recognition sites for ACT molecules, which recombined with the recognition sites in the membrane permeation experiment, ACT molecules penetration was hindered. However, the analogs of ECH successfully passed MIMs. It indicated that the selective MIMs for ACT followed the mechanism of delayed permeation. This work provides an important reference for the high permselective separation of natural products.

## Introduction

Phenylethanoid glycosides (PhGs) are the main active ingredients in *Cistanche tubulosa* (Wang et al., 2017; Yan et al., 2017), and the research have indicated that it has the functions of kidney-replenishing, anti-oxidation, anti-tumor, anti-aging, and improving memory (Wu C. J. et al., 2019; Xu et al., 2019a). Acteoside (ACT) and echinacoside (ECH) are the index composition of PhGs, and the structures of ACT and ECH are similar (Cui et al., 2016b; Morikawa et al., 2019; Wu C. J. et al., 2019). At present, the main separation and purification methods of PhGs are the chromatography, the membrane separation technique, and adsorption method (Han et al., 2012; Dong et al., 2015; Pei et al., 2019; Xu et al., 2019b). High-speed countercurrent chromatography has low yield and low efficiency. The disadvantages of adsorption methods are high solvent consumption and low yield. The advantages of membrane separation technology are high separation efficiency, low energy consumption, and strong applicability (Zhang et al., 2018; Hong et al., 2019; Ding et al., 2020; Sun et al., 2020). However, membrane separation technology has the problem of poor selectivity and low separation purity for natural products. Therefore, it was of great significance to improve the membrane selectivity.

Molecularly imprinted polymers (MIPs) are a kind of polymer with specific recognition and selective adsorption for specific target molecules (template molecules) and their structural analogs (Chen et al., 2016). MIPs are widely used in separation, drug delivery, catalysis, and sensing due to its specific recognition and selective adsorption functions (Yang et al., 2018). MIPs can make the target components of Chinese herbal medicine highly concentrated, almost free of impurities, and obtain high-purity products (Ma et al., 2019; Liang et al., 2020; Zhao et al., 2020a,b). However, molecularly imprinted polymers are easily lost during recovery and are not easy to industrialize (Huang et al., 2020; Li et al., 2020a,b; Zhang et al., 2020). Therefore, the development and design of a highly selective, durable, and recyclable separation membrane is called the research topic of separation of PhGs.

In recent years, studies have shown that the preparation of molecularly imprinted membranes (MIMs) by combining molecularly imprinted technology with membrane separation technology has made breakthroughs in molecular specific recognition, natural active substance separation, biomacromolecule separation, and chiral compound separation (Yoshikawa et al., 2016; Su et al., 2020). MIMs have the advantage of specific recognition of molecular imprints, the stability of membrane separation, and the advantage of reusability (Fu et al., 2019; Hassan et al., 2019; Yu et al., 2019). Today, chemical imprinting, surface modification, and physical blending are often used to prepare MIMs (Wu et al., 2018a; Gao et al., 2019; Lu et al., 2019; Wu Y. et al., 2019). However, the mechanical properties and stability of MIMs prepared by chemical grafting and physical blending methods are relatively low. Therefore, surface modification on the surface of commercial membranes is the most commonly used method for MIMs (Wu et al., 2018b; Zhao et al., 2018). The ultrafiltration membrane mainly includes a polyether sulfone membrane, a polyacrylonitrile membrane, a polyvinyl chloride membrane, and a polyvinylidene fluoride (PVDF) membrane. A PVDF membrane is a new and excellent membrane material with high mechanical strength, acid and alkali resistance, and good chemical stability (Cui et al., 2016a; Zhao et al., 2017). The glass transition temperature and melting point of PVDF membranes are −39 and 172°C, respectively. The thermal decomposition temperature of PVDF membranes is higher than 316°C. It indicated that PVDF membranes have a good thermal stability (Meng et al., 2016; Xia et al., 2019). The molecular linear structure of PVDF is relatively simple (-CH_2_CF_2_-), and the chemical bond energy of PVDF is high, and the bond energies of C-F, C-H, and C-C are 453, 414.5, and 347.5 kJ/mol, respectively. PVDF membrane exhibits excellent chemical stability (Chen et al., 2020). Thus, PVDF membranes are often used for biological and drug separation (Guo et al., 2019).

The main work of this study is to design and synthesize MIMs with high selectivity and high adsorption capacity for ACT. A series of different ACT-MIMs were synthesized by optimizing the types and amounts of functional monomers, crosslinking agents, and initiators. The surface characteristics and functional groups of the synthesized MIMs were characterized by SEM and FT-IR. The adsorption capacity and permeability of MIMs were evaluated by static binding experiments, and the binding selectivity of MIMs was studied. The enrichment and separation of ACT from the mixture of ACT and ECH by MIMs have achieved good results.

## Materials and Methods

### Materials and Reagents

The echinacoside (ECH, ≥98%) was supplied by the Shanghai Li Ding Biotechnology Co., Ltd. The acteoside (ACT, ≥98%) was purchased from the Shanghai Li Ding Biotechnology Co., Ltd. (Shanghai, China). 4-vinylpyridine (4-VP, 98%) and methacrylic acid (MAA, 98%) were purchased from the Aladdin Reagent Co., Ltd. (Shanghai, China). Ethyleneglycol dimethacrylate (EGDMA, 98%) was supplied by the Qingdao Ruinasi Polymer Material Co., Ltd (Qingdao, China), azodiisobutyronitrile (AIBN, 98%) was purchased from the Aladdin Reagent Co., Ltd. (Shanghai, China). *N, N*-dimethylformamide (DMF, 99.5%) was purchased from the Aladdin Reagent Co., Ltd. (Shanghai, China). Acrylamide (AM, 99%) was purchased from the Beijing Dingguo Biological Technology Co., Ltd. (Beijing, China). Acetonitrile (ACN, ≥99.9%) was supplied by Sinopharm Chemical, Reagent (Shanghai, China), Methanol (≥99.9%), and acetic acid (≥99.9%) were purchased from the Shanghai McLean Biochemical Technology Co., Ltd. (Shanghai, China). Ethanol (≥99.7%) was purchased from the Shanghai Shiyang Chemical Co., Ltd (Shanghai, China). Polyvinylidene fluoride membranes (PVDF membranes, 0.45 μm) were purchased from the RisingSun Membrane Technology Co., Ltd. (Beijing, China). Distilled water was supplied by the Smart-S15 system (Shanghai, China).

### HPLC Conditions

The e2695 fluid system (waters, USA) was used for analytical determination of ACT and ECH. A Symmetry C18 column (250 × 4.6 mm, 5 μm, reversed phase) was used to detect and separate the e2695 fluid system. Acetonitrile (A) and acetic acid/water (1:44, v/v) (B) were used as the mobile phase to inject at a flow rate of 1 mL/min, and the detection time was 40 min. The detection temperature of the column oven was 30°C. The detection wavelength of the ultraviolet detector was 330 nm. All samples were analyzed by retention time and compared with the UV-Vis spectrum of the standard.

### Characterization of Membranes

The surface morphology and microstructure of MIMs and NIMs were examined by SEM (ZEISS GeminiSEM 450, Carle Zeiss, Germany). The chemical structures of MIMs and NIMs were measured by the attenuation total reflection method using FT-IR (Nicolet iS50 FT-IR, Thermo Fisher Scientific, America). FT-IR scanning conditions: step size was 2 cm^−1^, and scanning range was 4,000–500 cm^−1^.

### MIMs Preparation

**Scheme 1:**
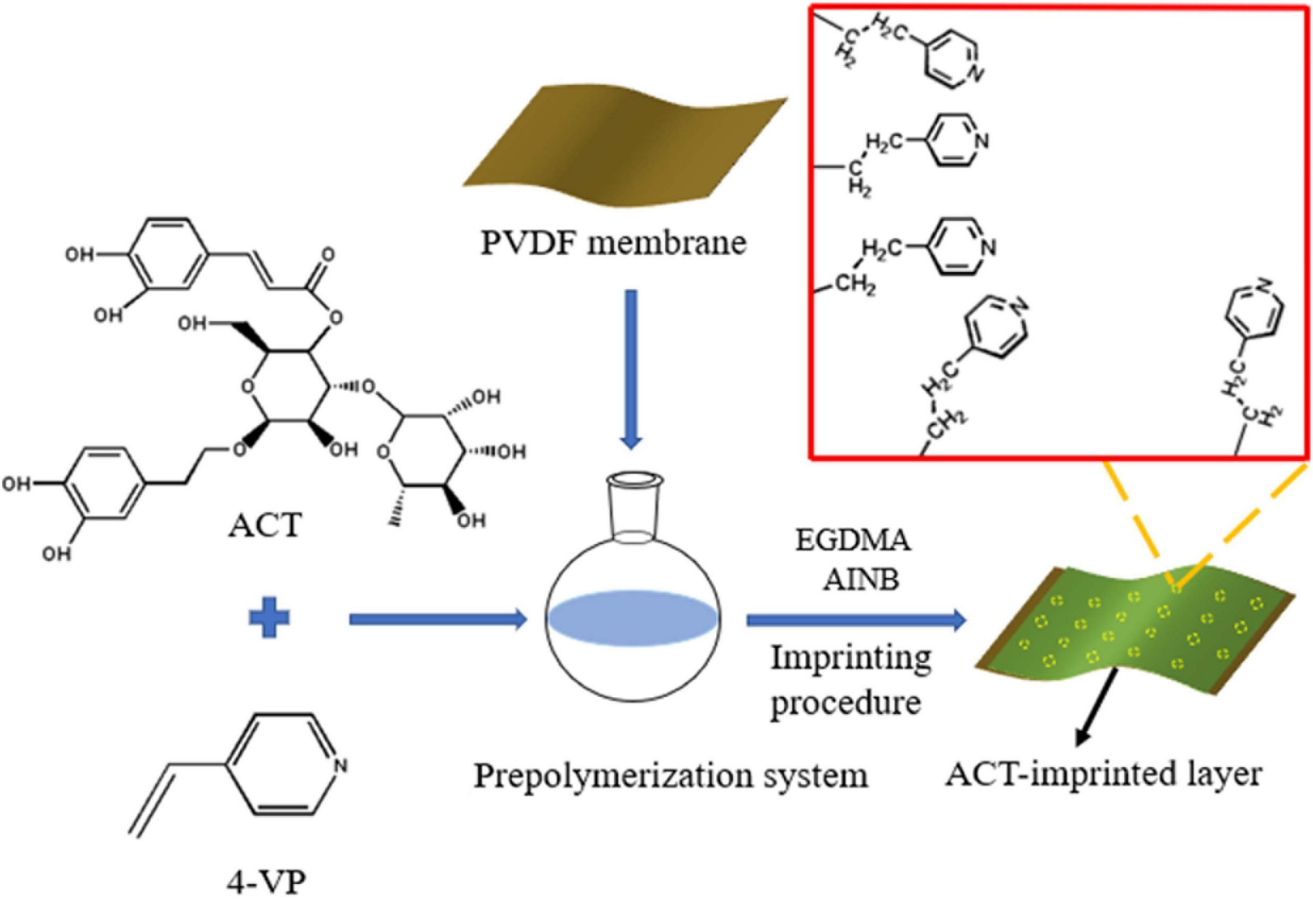
Schematic illustration of synthesis pathway of MIMs for selective separation of ACT.

According to Table 1, a series of MIMs (MIM1, MIM2, MIM12) were prepared by changing the type or quantity of functional monomers, crosslinkers, and initiators to explore the best preparation conditions. The preparation process was accomplished by *in-situ* polymerization with thermal initiation in a nitrogen atmosphere. The schematic diagram on the synthesis process of MIM is illustrated in Scheme 1. The whole preparation process is as follows:

**Table 1:** The preparation parameters of molecularly imprinted membrane.

**Membranes**	**Functional monomer (mmol)**			**Crosslinker (mmol)**	**Initiator (mmol)**
	**4-VP**	**AM**	**MAA**	**EGDMA**	**AIBN**
MIM1	2	-	-	6	0.1
MIM 2	-	2	-	6	0.1
MIM 3	-	-	2	6	0.1
MIM 4	4	-	-	6	0.1
MIM 5	6	-	-	6	0.1
MIM 6	8	-	-	6	0.1
MIM 7	6	-	-	4	0.1
MIM 8	6	-	-	8	0.1
MIM 9	6	-	-	10	0.1
MIM 10	6	-	-	4	0.2
MIM 11	6	-		4	0.3
MIM 12	6	-	-	4	0.4
NIM 7	6	-	-	4	0.1

*All MIMs were prepared by adding 0.2 mmol template molecules (ACT), and all NIMs were prepared without adding template molecules (ACT)*.

Firstly, a PVDF microfiltration membrane was immersed in methanol and activated for 24 h. Then rinsed with distilled water for 3 to 4 times to remove surface contamination which allowed the PVDF membrane to expand moderately. Following that, the PVDF membrane was immersed in 0.05 mol/L mixed solution of AIBN and methanol for 10 min. After this, it was placed in the oven to dry at 40°C to drain the excess membrane solution.

Secondly, a mixed solution of acetonitrile and *N, N*-dimethylformamide was prepared at a volume ratio of 1:1.5, and 2.5 mL of the mixed solution was taken to dissolve 125.00 mg ACT and 210.00 mg 4-VP. Then, the mixture was prepolymerized at 25°C for 2 h.

Subsequently, 1.12 g EGDMA and 16.40 mg AIBN were added and completely dissolved in the prepolymerization solution. Then, the PVDF membrane was immersed into the resulting casting solution and reacted in a nitrogen atmosphere for 23 min.

Finally, the excess casting fluid was expelled from the membrane surface. The membrane was placed in an oil bath at 60°C under a nitrogen atmosphere and thermally initiated for 24 h to complete the polymerization.

The template molecule ACT in the imprinted layer of MIMs was eluted with a mixed solution of methanol and acetic acid with a volume ratio of 9:1 until there was no ACT in the eluent. Then, the acetic acid on the MIMs was removed with methanol to obtain MIMs with template molecules removed and stored in methanol. The difference between non-molecularly imprinted membranes (NIMs) and MIMs is that no template molecules were added during the synthesis.

### Static Binding Experiments

The adsorption capacity of the membrane is an important indicator to evaluate the performance of the membrane. Therefore, it is necessary to analyze the adsorption capacity of the membrane through static binding experiments. In the isothermal adsorption experiment, a piece of MIMs or NIMs was immersed in 20 mL ACT solution of different concentrations (0.20, 0.40, 0.60, 0.80, and 1.00 mg/mL) and placed in constant temperature for adsorption (30°C, 24 h). In the adsorption kinetics experiment, a piece of MIMs or NIMs was immersed in 20 mL of ACT solution with an initial concentration of 0.5 mg/mL for adsorption (30°C, 24 h). Samples were taken at different time points (0.5 mL), and the concentration of ACT in the solution at different time points was determined by HPLC. The binding capacities of MIM7 and NIM7 were calculated according to the formula as follows (Fan et al., 2014; Yao et al., 2018):

(1) $$\begin{array}{r} B_{t}=\frac{\left(C_{0}-C_{t}\right)V}{m} \end{array}$$

(2) $$\begin{array}{r} B_{e}=\frac{\left(C_{0}-C_{e}\right)V}{m} \end{array}$$

where *B*_*t*_(mg/g) and *B*_*e*_ (mg/g) represent the ACT adsorption amount at time *t* and equilibrium adsorption amount, respectively. *C*_*t*_ and *C*_*e*_ are the concentrations (mg/mL) at time *t* and equilibrium concentration, respectively. *C*_0_ is the initial ACT concentration; *V* is the volume (mL) of the solution; and *m* is the mass (g) of MIMs or NIMs.

In addition, in order to evaluate the selective adsorption performance of MIMs or NIMs, the prepared MIMs or NIMs were immersed in a mixture containing ACT and its structural analog ECH (the initial concentration of each substance was 0.5 mg/mL) for adsorption (30°C, 24 h). The selectivity factor (α) was determined as (Gao et al., 2019):

(3) $$\begin{array}{r} \alpha =\frac{B_{ACT}/C_{ACT}}{B_{ECH}/C_{ECH}} \end{array}$$

where *B*_*ACT*_ (mg/g) and *B*_*ECH*_ (mg/g) represent the equilibrium adsorption amount of MIMs or NIMs to ACT and ECH, respectively. *C*_*ACT*_ and *C*_*ECH*_represent the equilibrium concentrations of MIMs or NIMs to ACT and ECH, respectively.

### Membrane Permeation Measurement

Permeability selectivity is an important evaluation index for the selective separation performance of MIMs. In the osmosis experiment, a mixed solution consisting of ACT and ECH (0.5 mg/mL) was used to evaluate the relationship between osmotic selectivity and the change between them. In order to evaluate the selective recognition characteristics of MIM for ACT, a permeation experiment was carried out in an H-shaped permeation tank. The cross-section diameter of the opening of the H-shaped permeation tank was 1.5 cm. The membrane was installed on the connection port of the two permeation cells and the connection port was sealed (there is no pressure difference between them). The permeate side of the H-shaped permeation tank as the donor chamber received 50 mL of the initial concentration of 0.5 mg/mL of ACT/ECH. The other side of the H-shaped permeation tank as the acceptor chamber received an equal volume of water. The solution was stirred with magnetic force in the osmosis cells on both sides, and then samples were taken from the solution at a certain time point. The concentration of ACT and ECH in the solution was determined by HPLC. Permselectivity factor β, permeability coefficient *P* (cm^2^/s), and permeation flux *J* (mg/cm^2^·s) were calculated by the following equations (Wu et al., 2014, 2015):

(4) $$\begin{array}{r} J_{X}=\frac{\Delta C_{X}V}{\Delta tA} \end{array}$$

(5) $$\begin{array}{r} P=\frac{J_{X\;}d}{\left(C_{FX}-C_{RX}\right)} \end{array}$$

(6) $$\begin{array}{r} \beta_{i/j}=\frac{P_{i}}{P_{j}} \end{array}$$

where *A, d*, and *V* represent the effective areas of the membrane (cm^2^), the thickness of membranes (cm), and the solution volume of donor or acceptor chamber (mL), respectively. Δ*C*_*X*_*/*Δ*t* is the rate of change of the concentration of the solution in the acceptor chamber. *(C*_*FX*_*-C*_*RX*_*)* is the concentration difference of the solution on both sides of the osmosis cell. *X* is the target ACT or analog ECH. *i* and *j* are the target ACT and the analog ECH, respectively.

## Results and Discussion

### Optimization of Synthesis Conditions

**Figure 1:**
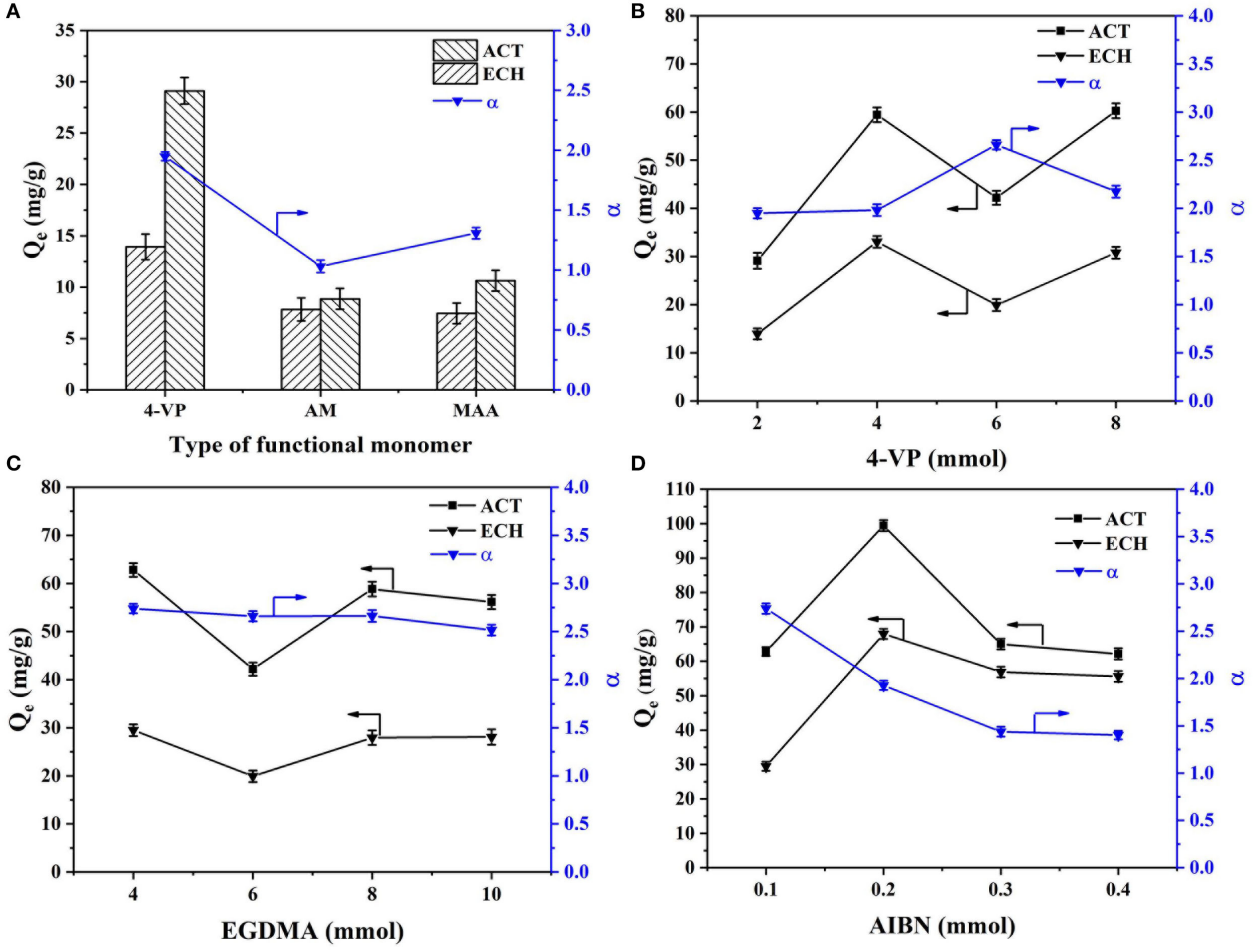
Effects of the **(A)** type of functional monomer, **(B)** amount of functional monomer, **(C)** amount of crosslinking agent, and **(D)** amount of initiator on rebinding the capacity of MIMs at 30°C.

In order to explore the optimal synthesis conditions, the preparation conditions (type of functional monomer, amount of functional monomer, amount of crosslinking agent, and amount of initiator) were optimized and discussed. The effects of the type of functional monomer, amount of functional monomer, amount of crosslinking agent, and amount of initiator on rebinding the capacity of MIMs at 30°C are shown in Figure 1.

As shown in Figure 1A, MIMs prepared with different functional monomers showed different rebinding ability. The rebinding ability and selectivity of MIM1 prepared with 4-VP as the monomer were higher than those prepared with AM and MAA as the monomers. The rebinding ability of MIM1 prepared with 4-VP as the monomer to ACT and ECH were 29.11 mg/g and 13.93 mg/g, respectively. The selectivity factor of MIM1 was 1.95. The ACT molecule was an acidic imprinted molecule, and 4-VP as the basic functional monomer was selected, which was conducive to the formation of a stable host and guest complexes of the template molecule and functional monomer. However, AM and MAA were a neutral and acidic functional monomer respectively, which were not conducive to the formation of stable hydrogen bonds of the template molecule and functional monomer (Omran et al., 2019). Therefore, 4-VP as a functional monomer can facilitate MIMs recombination and selective separation of ACT.

As shown in Figure 1B, the amount of functional monomer plays a key role in selectivity of MIMs. When the amount of 4-VP was 6 mmol, the selectivity factor of MIM5 was 2.66, and the adsorption capacity of ACT and ECH were 42.19 mg/g and 19.92 mg/g (Figure 1B), respectively. As the amount of 4-VP was small, only a small fraction of ACT can bind to 4-VP. The excess ACT was in free state, which leads to the low amount of imprinted holes of ACT. Increasing the amount of the functional monomer 4-VP can make the preassembly between imprinted molecules and functional monomers more full. However, if the amount of 4-VP was too large, the self-association of 4-VP will occur, which will reduce the binding sites in the imprinted layer of MIMs (Mao et al., 2016).

As shown in Figures 1C,D, the amount of crosslinking agent and initiator will affect the selectivity and adsorption of MIMs. As the amount of crosslinking agent and initiator increases, the selectivity of MIMs will gradually decrease. This is because a high amount of crosslinking agent and initiator will lead to a high crosslinking degree of polymer in the MIMs imprinted layer, making the accessibility of imprinted sites worse. Thus, the selectivity of MIMs will be reduced.

Through the optimization and discussion of the type of functional monomer, the amount of functional monomer, the amount of crosslinking agent, and initiator, it can be concluded that the performance of MIM7 was the best. The optimal synthesized conditions of MIMs were synthesized by using ACT as the template molecule, 4-VP as the functional monomer, EGDMA as the crosslinking agent, AIBN as the initiator, and their ratio was 2:60:40:1. The selectivity factor of MIM7 was 2.74, and the adsorption capacity of ACT and ECH were 62.83 and 29.49 mg/g, respectively.

### Characterizations of Membranes

**Figure 2:**
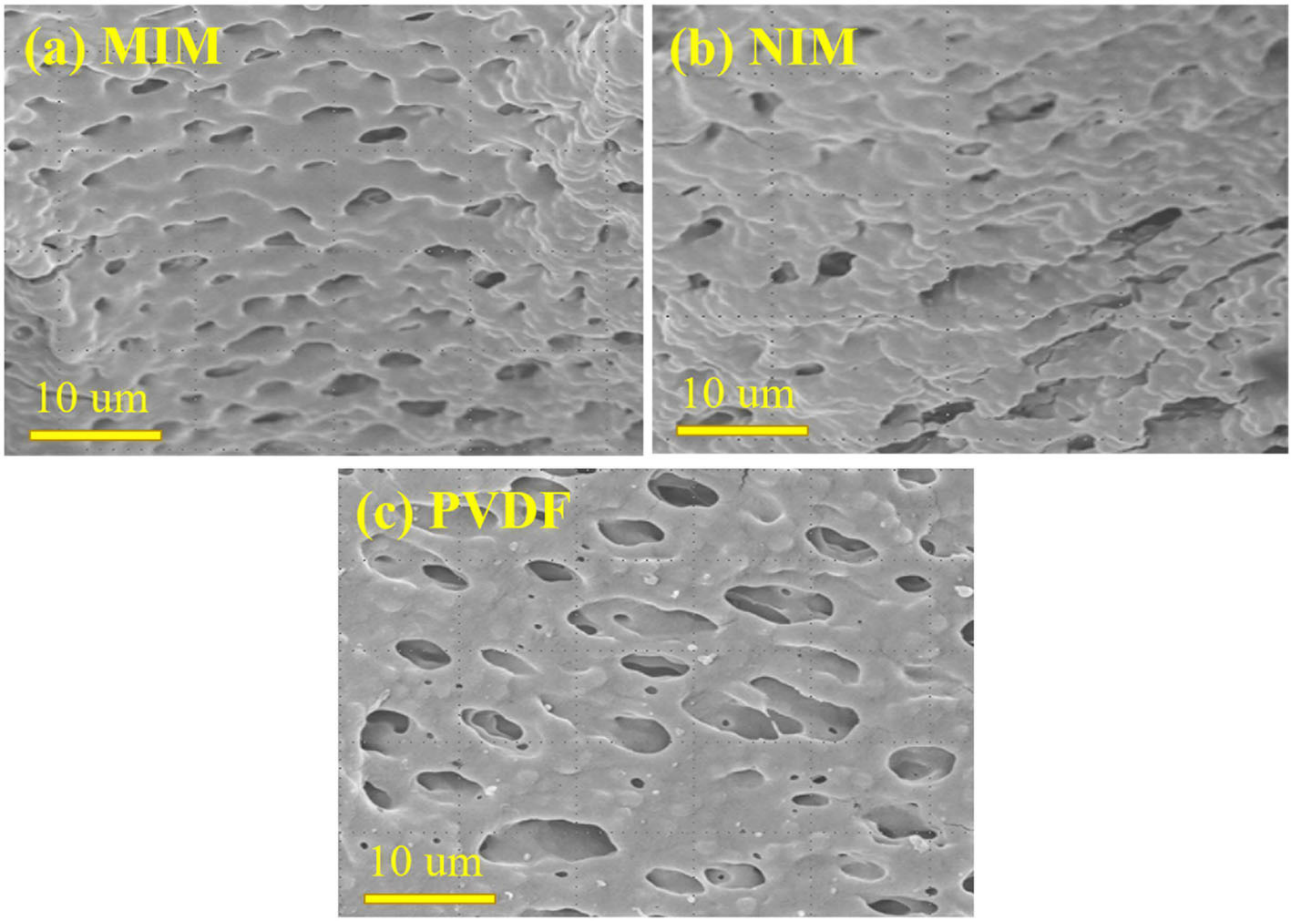
SEM of **(A)** MIM7, **(B)** NIM7, and **(C)** PVDF.

The surface morphology and microstructure of MIM7, NIM7, and PVDF were shown in Figure 2. Figure 2A shows the surface of the MIM7 uniform distribution of the 2 μm imprinting holes. As shown in Figure 2B, the surface of the NIM7 is distributed with a few uneven holes. As shown in Figure 2C, the surface of the PVDF membrane has ~10 μm holes. Compared with the PVDF membrane, it can be seen that the pores on the surface of MIM7 and NIM7 are smaller. It indicated that the imprinted layer was successfully synthesized on the surface of the PVDF membrane.

**Figure 3:**
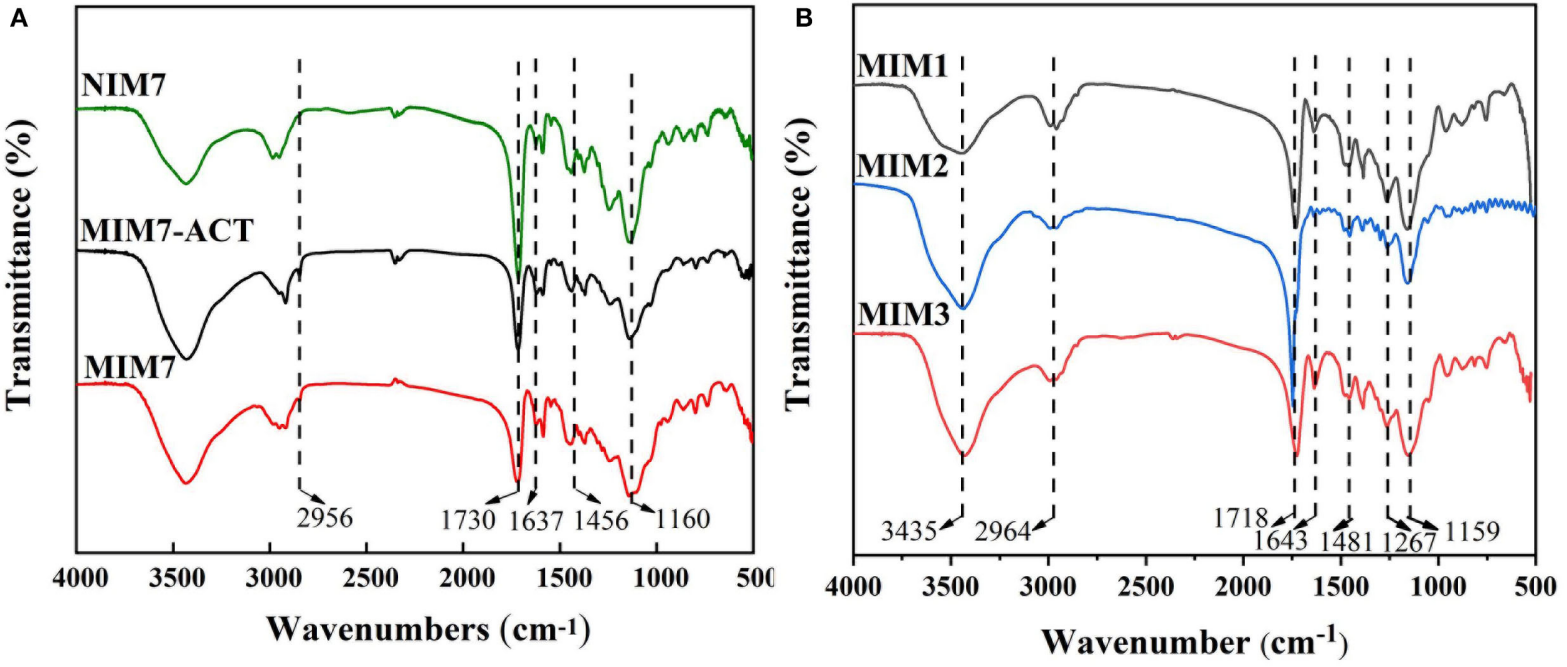
FT-IR spectra of **(A)** NIM7, MIM7-ACT, and MIM7, **(B)** MIM1, MIM2, and MIM3.

Figure 3A is the FT-IR spectra of NIM7, MIM7-ACT, and MIM7. The peaks at around 1,637 and 1,456 cm^−1^ represented the bending vibrations of C = N and C = C from 4-VP, respectively. From the spectrum analysis, it is concluded that MIM7 and NIM7 had been successfully prepared. NIM7 and MIM7 had the same characteristic peak, it indicated that their chemical structures were similar. By comparing the spectra of MIM7 and MIM7-ACT, it can be found that MIM7-ACT had a new peak at 2,956 cm^−1^, which was due to the hydrogen bonding association between ACT and 4-VP on MIM7. It indicated that the template molecule ACT and MIM7 have been recombined through hydrogen bonding.

Figure 3B is the FT-IR spectra of MIM1, MIM2, and MIM3. The peaks at around 1,643 and 1,481 cm^−1^ can be attributed to the bending vibrations of C = N and the stretching vibrations of C = C from 4-VP, respectively. The peaks at around 1,643 and 1,718 cm^−1^ represented the tensile vibration of C = N and C = O from AM. The peak at 1,643 cm^−1^ corresponded to the tensile vibration of C = O from MAA. It suggested that 4-VP, AM, and MAA were successfully introduced into the MIMs.

### Binding Analysis and Recognition Specificity of MIM7

In order to conduct a detailed evaluation of the recombination capability of MIM7, a system static adsorption experiment is required. Firstly, the isothermal adsorption experiments were carried out on MIM7 and NIM7, and analyzed through mathematical models. Secondly, the adsorption kinetics of MIM7 and NIM7 and their rate control components were studied.

**Figure 4:**
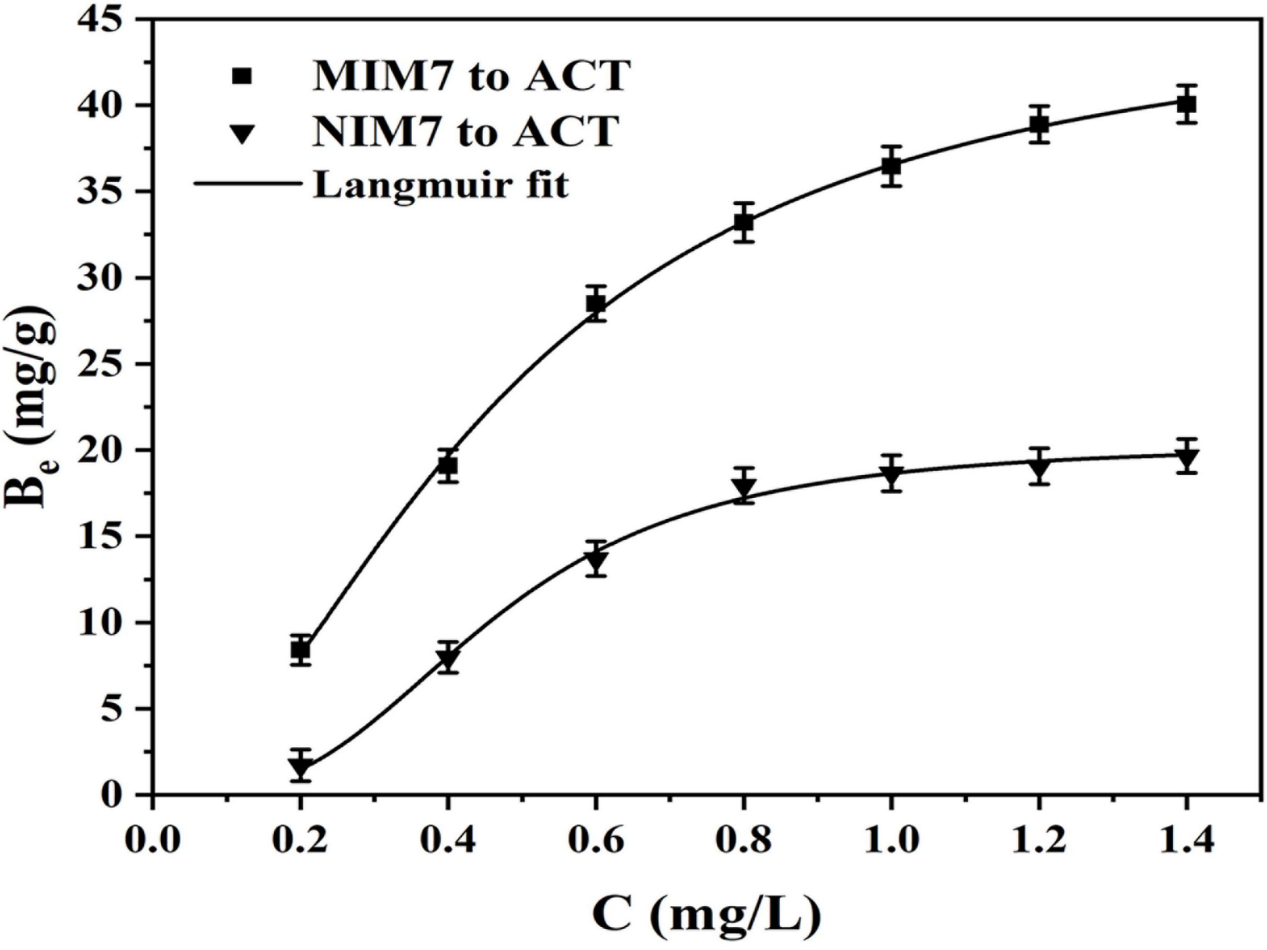
Equilibrium data and Langmuir modeling for the adsorption of ACT onto MIM7 and NIM7.

Figure 4 shows the adsorption equilibrium data and Langmuir model of ACT on MIM7 and NIM7. The Langmuir adsorption isotherm constants for ACT on MIM7 and NIM7 have been listed in Table 2. It can be seen from Figure 4 that the rebinding ability of MIM7 and NIM7 to bind ACT molecules is closely related to the initial concentration of ACT molecules, and this recombination ability also increases as the initial concentration increases. Moreover, MIM7 has a higher rebinding ability to ACT than NIM7, this due to MIM7 having more imprinted holes and high affinity sites in the molecularly imprinted layer. In particular, when the initial concentration was 1.4 mg/mL, the rebinding ability of MIM7 was much higher than that of NIM7 (the recombination amount of MIM7 is 40.06 mg/g, and the recombination amount of NIM7 is 19.66 mg/g). In addition, the Langmuir isotherm adsorption model was established to fit the experimental data, the linear regression value (*R*^2^) was 0.9986, and the adsorption mechanism of MIM7 was further studied. It indicated that MIM7 rebinding sites were evenly distributed on the surface, while the adsorption to ACT was considered as monolayer adsorption.

**Table 2:** Langmuir adsorption isotherm constants for ACT onto the MIM7 and NIM7.

**Membranes**	***Q_***e,exp***_* (mg g^**−1**^)**	***Q_***e,c***_* (mg g^**−1**^)**	***K_***L***_*(L mg^**−1**^)**	***R^**2**^***
MIM7	40.0615	45.8685	0.0039	0.9986
NIM7	19.6581	20.3847	0.0080	0.9961

**Figure 5:**
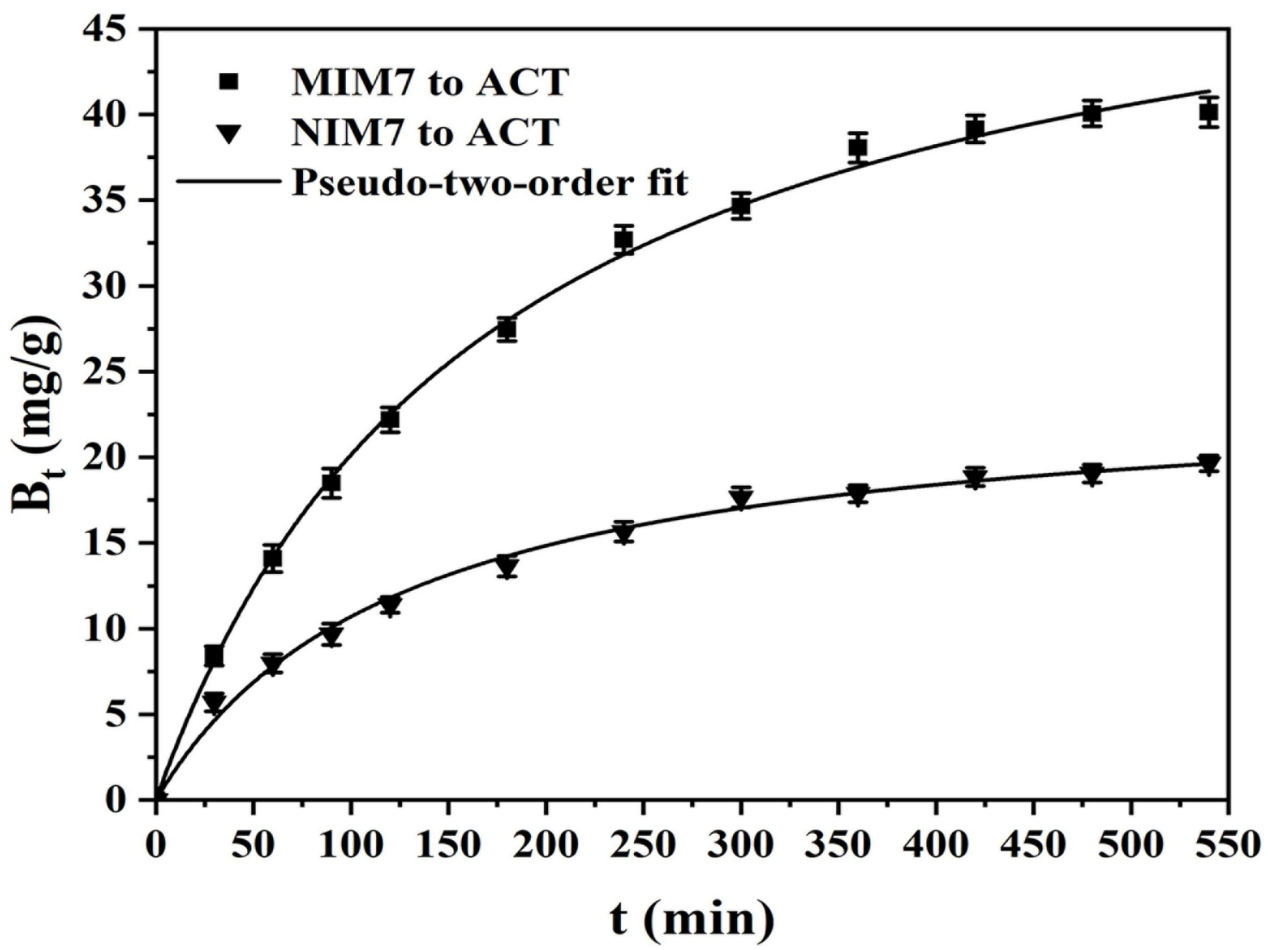
Kinetic curves and fitting model of ACT onto MIM7 and NIM7.

Figure 5 shows the kinetic curves and fitting model of ACT onto MIM7 and NIM7. Table 3 shows kinetics constants for the pseudo-first-order and pseudo-second-order rate equations. The dynamic adsorption of MIM7 was performed in 0.50 mg/mL ACT solution. From the data trend in the Figure 5, it can be seen that the adsorption capacity of ACT by MIM7 increases rapidly from 0 to 240 min, and increases slowly from 240 to 420 min, but almost unchanged after 420 min. Therefore, the adsorption of MIM7 reached equilibrium at 420 min, and the equilibrium adsorption amount was 40.08 mg/g. From 0 to 240 min, the ACT solution has more free ACT molecules, and the imprint holes in MIM7 are not combined, so the hydrogen bonding speed of MIM7 and ACT is faster. Subsequently, as the amount of rebinding of MIM7 and ACT molecules increases, the recognition sites on MIM7 and the content of ACT in the solution were reduced, so that the recombination rate was reduced until the dynamic equilibrium was reached. In addition, the adsorption processes of MIM7 and NIM7 were fitted with kinetic models. It can be seen from the linear fitting results in Table 3 that the experimental data were consistent with the pseudo-second-order dynamic fitting data. The linear regression *R*^2^ of MIM7 and NIM7 were 0.9993 and 0.9983, respectively. The dynamic adsorption equilibrium data were basically consistent with the pseudo-second-order *r* kinetic fitting data, which shows that the chemical adsorption of MIM7 to ACT is dominant. Therefore, it indicated that this process of adsorption behavior may be hydrogen bonding.

**Table 3:** Kinetics constants for the pseudo-first-order and pseudo-second-order rate equations.

**Membranes**		**Pseudo-first-order model**			**Pseudo-second-order model**		
	***Q_***e,exp***_* (mg g^**−1**^)**	***Q_***e,c***_* (mg g^**−1**^)**	***K_**1**_* (L mg^**−1**^)**	***R^**2**^***	***Q_***e,c***_* (mg g^**−1**^)**	***K_**2**_* (L mg^**−1**^)**	***R^**2**^***
MIM7	40.1232	30.4442	0.0017	0.7184	53.4141	0.0011	0.9993
NIM7	19.6581	14.4721	0.0012	0.8134	24.2110	0.0032	0.9983

### Selective Transport and Separation Mechanism of MIM7

In order to gain a deeper understanding of the separation performance of MIM7 and NIM7, the permselectivity experiment was required. Permselectivity was measured at a constant temperature of 30°C, and the initial concentration of the permeate was 0.5 mg/mL. In the experiment, ACT and ECH were used as competing molecules to study the time-dependent penetration selectivity results of MIM7 and NIM7.

**Figure 6:**
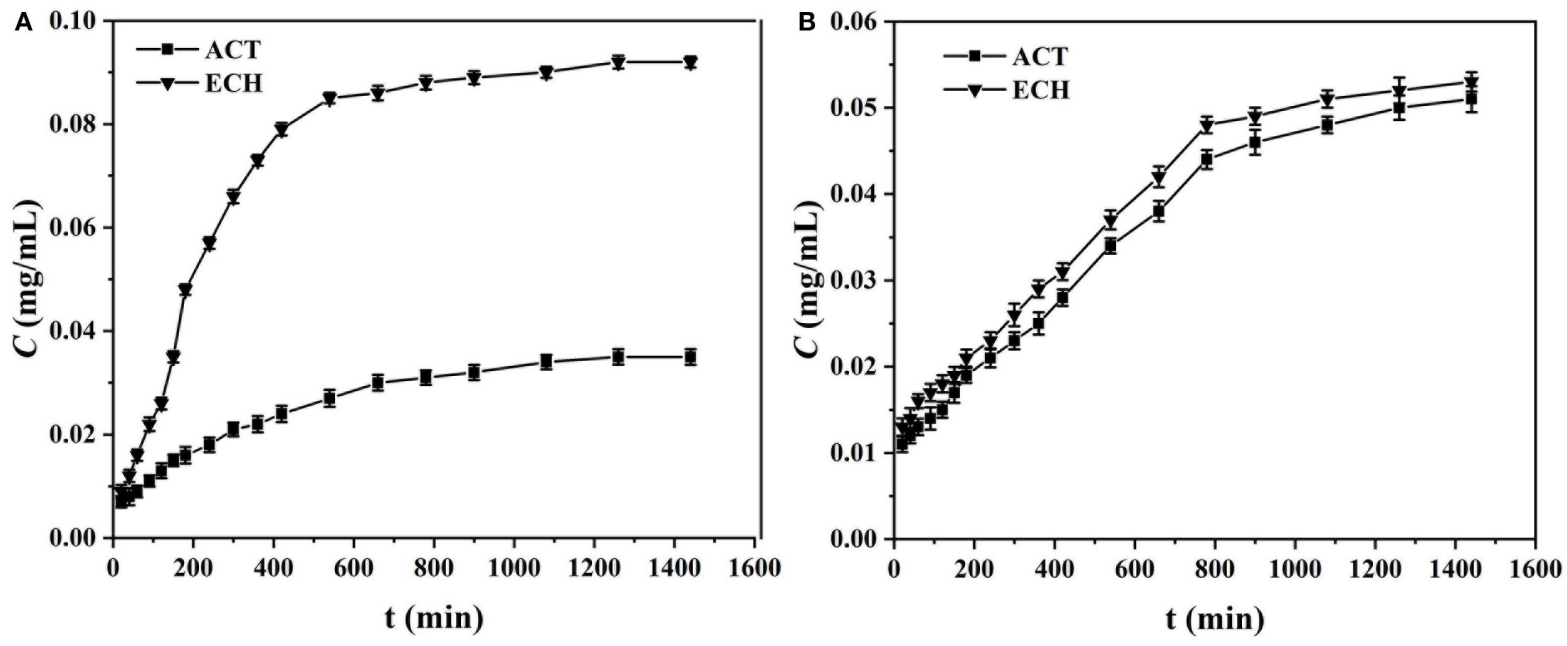
Time-dependent permselectivity curves of various targets (ACT and ECH) through **(A)** MIM7 and **(B)** NIM7.

Figure 6 is the time-dependent permselectivity curves of two target molecules (ACT and ECH) through MIM7 and NIM7. As shown in Figure 6A, the concentration of the solution on the left and right sides of the MIM7 permeation pool reached 800 min to equilibrium, and the equilibrium concentration of ECH and ACT in the receiving pool were 0.092 and 0.035 mg/mL, respectively. As shown in Figure 6B, the concentration of the solution on the left and right sides of the NIM7 permeation pool reached 800 min to equilibrium, and the equilibrium concentration of ECH and ACT in the receiving pool were 0.092 and 0.035 mg/mL, respectively. The presence of ACT imprinted holes on MIM7 results in the reorganization of ACT and MIM7, which hinders the transfer of ACT molecules. However, there were no imprinted holes of ECH on MIM7, and ECH would not recombine during transmission. Therefore, ECH passed MIM7 more easily. In contrast, NIM7 had no imprinted holes of ACT and ECH, so ACT and ECH had almost the same penetration. It indicated that there was no imprinting site in NIM7. More importantly, the permselectivity and separation factors (β_*ACT*/*ECH*_) data were mainly in Table 4, where the β_*ACT*/*ECH*_ values of MIM7 are all >2.66. This clearly showed that the excellent selective separation performance of MIM7 and the presence of specific recognition sites for ACT only.

**Table 4:** Time-permeation results (800 min) of MIM7 and NIM7 for ACT and ECH.

**Membranes**	**Substrates**	**J (mg·cm^−2^·h^−1^)**	**P (cm^2^·h^−1^)**	***β_*ACT*/*ECH*_***
MIM7	ACT	1.93 × 10^−5^	4.02 × 10^−6^	2.66
	ECH	5.06 × 10^−5^	1.07 × 10^−5^	
NIM7	ACT	1.40 × 10^−5^	2.92 × 10^−6^	1.04
	ECH	1.46 × 10^−5^	3.05 × 10^−6^	

The existing permeation selectivity mechanisms of MIMs are mainly two different mechanisms for promoting penetration and delaying penetration (Wu et al., 2018b; Wu Y. et al., 2019). According to the above penetration experiment results, it can be seen that the ACT molecule has not passed MIM7, and the ECH has passed smoothly. This indicates that delayed penetration plays a dominant role in the selective separation of MIM7. The separation efficiency of ACT mainly depends on the imprint holes of MIM7. In other words, this is because the presence of holes imprinted on the surface of MIM7 makes ACT molecules recombine with MIM7, while in ECH this does not occur (Wang et al., 2015). Therefore, ECH will directly pass through MIM7 during the recombining process.

### Regeneration and Stability of MIM7

**Figure 7:**
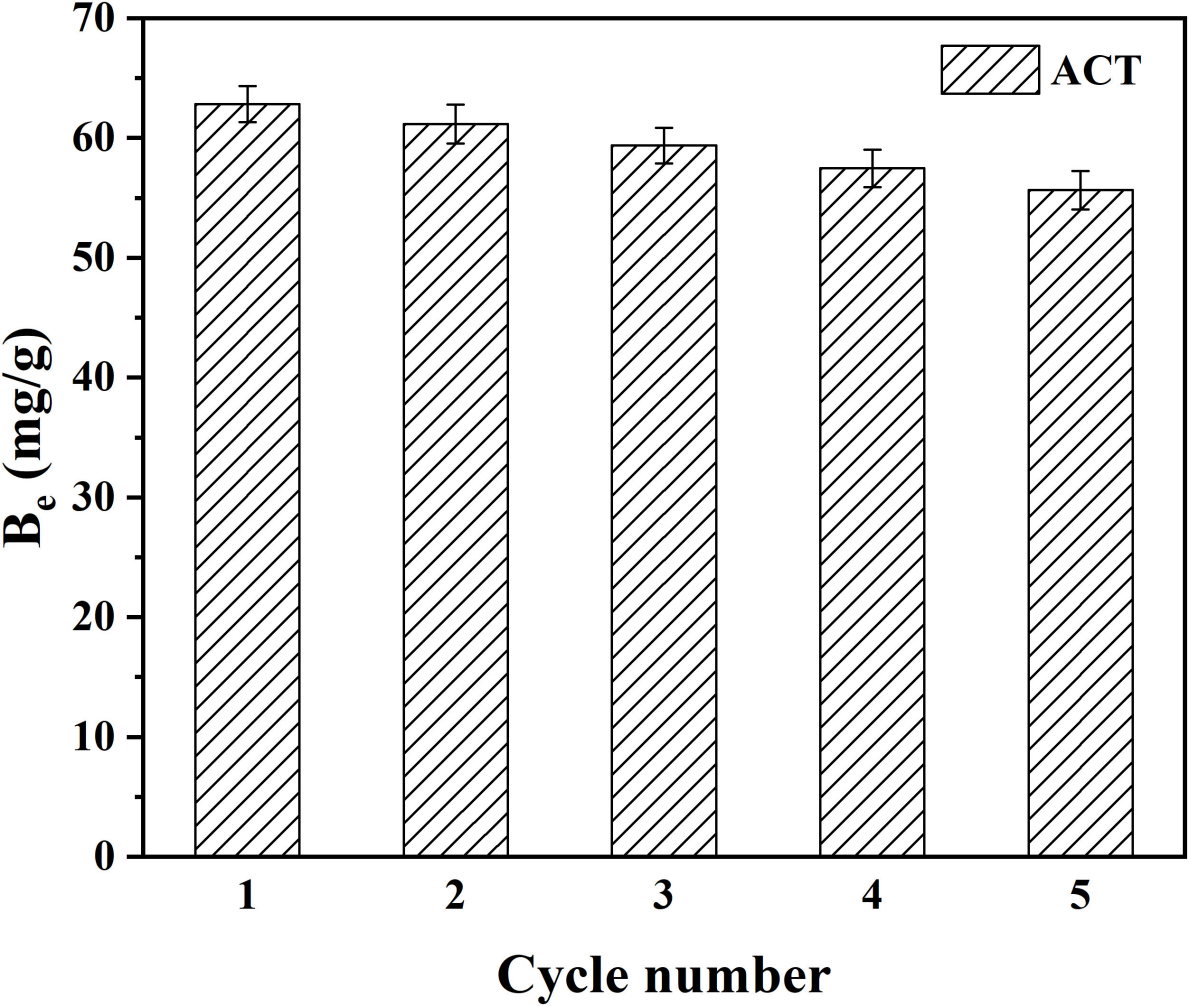
Adsorption-desorption cycles of MIM7 for ACT adsorption.

It is well-known that the circularity and stability of MIMs are a very critical and important property. Therefore, the adsorption stability and regeneration performance of MIM7 were studied by the adsorption/desorption cycle experiment of MIM7. As shown in Figure 7, the initial adsorption capacity of MIM7 was 62.83 mg/g, and the adsorption capacity gradually decreased as the number of cycles increased. When the fifth cycle was reached, the adsorption capacity of MIM7 was 55.65 mg/g, and it was 88.57% of the maximal adsorption amount. It indicated that MIMs based on PVDF had a good stability.

## Conclusions

In summary, a kind of ACT-MIMs based on a PVDF membrane was developed for the selective separation of ACT. The optimal MIMs were prepared by investigating the types of functional monomers, the amounts of functional monomers, the amounts of crosslinking agent, and the amounts of initiator. The optimal synthesized conditions of MIMs were prepared by using ACT as a template molecule, 4-VP as a functional monomer, EGDMA as a crosslinking agent, and AIBN as initiator, and their molecular ratio was 2:60:40:1. The separation performance of MIM7 was evaluated by an adsorption and permeation test. The MIM7 prepared under optimal preparation conditions showed excellent performance. The selectivity factor was 2.74 and the permeability coefficient exceeded 2.66. According to the phenomenon of penetration experiment and data analysis, the permeation mechanism of MIMs delayed permeation. It indicated that the MIMs have high recognition rate and good stability. This work has a significant guidance role for the separation of ACT and its structural analogs, and MIMs exhibited a potential application in the separation of natural products.

## Data Availability Statement

All datasets generated for this study are included in the article/supplementary material.

## Author Contributions

XZ carried out the experiments and wrote the manuscript. YC, HX, and YH assisted in the collection of experimental data. YL contributed to the study design. XL contributed to the study design, manuscript revision, and final version. All authors contributed to the article and approved the submitted version.

## Conflict of Interest

The authors declare that the research was conducted in the absence of any commercial or financial relationships that could be construed as a potential conflict of interest.
